# Mosquito-borne Inkoo virus in northern Sweden - isolation and whole genome sequencing

**DOI:** 10.1186/s12985-017-0725-5

**Published:** 2017-03-23

**Authors:** Olivia Wesula Lwande, Göran Bucht, Clas Ahlm, Kristoffer Ahlm, Jonas Näslund, Magnus Evander

**Affiliations:** 1 Department of Clinical Microbiology, Virology, Umeå University, Umeå, Sweden; 2 Swedish Defence Research Agency, CBRN Defence and Security, Umeå, Sweden; 3 Department of Clinical Microbiology, Infectious Diseases, Umeå University, Umeå, Sweden

## Abstract

**Background:**

Inkoo virus (INKV) is a less known mosquito-borne virus belonging to *Bunyaviridae*, genus *Orthobunyavirus*, California serogroup. Studies indicate that INKV infection is mainly asymptomatic, but can cause mild encephalitis in humans. In northern Europe, the sero-prevalence against INKV is high, 41% in Sweden and 51% in Finland. Previously, INKV RNA has been detected in adult *Aedes (Ae.) communis, Ae. hexodontus* and *Ae. punctor* mosquitoes and *Ae. communis* larvae, but there are still gaps of knowledge regarding mosquito vectors and genetic diversity. Therefore, we aimed to determine the occurrence of INKV in its mosquito vector and characterize the isolates.

**Methods:**

About 125,000 mosquitoes were collected during a mosquito-borne virus surveillance in northern Sweden during the summer period of 2015. Of these, 10,000 mosquitoes were processed for virus isolation and detection using cell culture and RT-PCR. Virus isolates were further characterized by whole genome sequencing. Genetic typing of mosquito species was conducted by cytochrome oxidase subunit I (COI) gene amplification and sequencing (genetic barcoding).

**Results:**

Several *Ae. communis* mosquitoes were found positive for INKV RNA and two isolates were obtained. The first complete sequences of the small (S), medium (M), and large (L) segments of INKV in Sweden were obtained. Phylogenetic analysis showed that the INKV genome was most closely related to other INKV isolates from Sweden and Finland. Of the three INKV genome segments, the INKV M segment had the highest frequency of non-synonymous mutations. The overall G/C-content of INKV genes was low for the N/NSs genes (43.8–45.5%), polyprotein (Gn/Gc/NSm) gene (35.6%) and the RNA polymerase gene (33.8%) This may be due to the fact that INKV in most instances utilized A or T in the third codon position.

**Conclusions:**

INKV is frequently circulating in northern Sweden and *Ae. communis* is the key vector. The high mutation rate of the INKV M segment may have consequences on virulence

**Electronic supplementary material:**

The online version of this article (doi:10.1186/s12985-017-0725-5) contains supplementary material, which is available to authorized users.

## Importance

Inkoo virus (INKV) is endemic in northern Europe and may cause an influenza-like illness that may develop to a more severe form with encephalitic symptoms. Interestingly, the sero-prevalence is unexpectedly high among human populations in Finland and Sweden. In endemic areas, mammals have also been shown to have specific antibodies against INKV and the mountain hare (*Lepidus timidus*) is speculated to be a reservoir host of INKV. Previous findings of INKV infections in human populations of northern Europe, and the possibility of the virus to be vertically transmitted by mosquitoes in endemic areas, highlights the need for further surveillance and characterization of virus strains to elucidate the impact of INKV infections. In this study, INKV was isolated from *Ae. communis* mosquitoes, and the full genome sequences of the three segments were obtained. In addition, further identification of mosquito vectors that may transmit INKV is necessary in the establishment of appropriate vector control, surveillance and infectious disease modelling.

## Background

Most mosquito-borne viruses belong to three major families; *Bunyaviridae, Flaviviridae* and *Togaviridae* [1, 2]. Most of these viruses have been associated to cause high morbidity and mortality resulting in massive economic loss worldwide [3, 4]. In Europe, ten mosquito-borne viruses are known to be pathogenic to humans. Some are most probably endemic, i.e. Batai virus, Inkoo virus (INKV), Sindbis virus (SINV), Snowshoe hare virus, Chatanga virus [5], Tahyna virus, Usutu virus and West Nile virus; while some occur sporadically i.e. dengue- and chikungunya virus [2].

INKV belongs to family *Bunyaviridae*, genus *Orthobunyavirus*, California serogroup and was first isolated in 1964 from *Ae. communis* in Inkoo, Finland [6]. Since then, subsequent isolations of the virus have been reported in, Russia, Norway and Sweden [7–9]. Recently, we detected and sequenced INKV RNA present in *Aedes* (*Ae.*) *communis* larvae in northern Sweden [10], which suggests possible vertical transmission by mosquitoes. It is not fully known whether there are other INKV competent mosquitoes. In Sweden, there are around 50 known blood-sucking mosquito species of which more than 16 species are present in the northern part of the country [11, 12]. The proportion of different mosquito species varies over Sweden especially during the summer season where *Ae. communis* is the most abundant species in the north during the first half of the summer [12].

The clinical picture of INKV disease is not fully known. It is assumed that most infected persons are asymptomatic or present a mild febrile illness, which is self-limiting in most cases. However, in severe cases, it may cause a neuroinvasive disease that manifest in a form of encephalitis [5, 13]. The sero-prevalence of INKV is high among human populations in endemic areas of Finland (51%) and Sweden (41%) [14, 15].

INKV has a tri-segmented, negative-sense, single-stranded RNA genome that is approximately 12 kb in size. The S segment (1 kb) encodes the nucleocapsid protein (N), the M segment (4.5 kb) encodes for a polyprotein that is subsequently cleaved into two envelope glycoproteins Gn and Gc and the L segment (6.9 kb) encodes the RNA-dependent RNA polymerase [16, 17]. In addition, several viruses of the *Bunyaviridae* family encode nonstructural NSs and NSm proteins from the S and M segments, respectively.

Tri-segmented mosquito-borne viruses from the *Orthobunyavirus* genus, Bunyamwera sero-group can undergo genetic re-assortments that possibly occur during co-infection [18–20]. For instance, Ngari virus is known to be a Bunyamwera re-assortant. Its S and L segments belong to Bunyamwera virus, while the M-segment belongs to Batai virus [18]. Genetic analyses of the Tahyna virus M-segment indicate high variability, especially in the Gc and NSm genes [21]. Ngari- virus and Tahyna virus belong to same genus, *Orthobunyavirus,* but are classified in distinct serogroups; Bunyamwera and California respectively. The ability of La Crosse virus (a virus belonging to the same serogroup as INKV) to undergo reassortment in nature via vertical transmission was demonstrated by rearing mosquito eggs to adults and the virus RNA isolated from their abdomen. Analyses of the S, M and L of the La Crosse virus sequences, indicated that about a quarter of the infected mosquitoes contained reassorted genome segments [22]. Sequence comparison of 56 Jamestown Canyon virus strains in a span of 40 years in Connecticut indicate divergent evolutionary history of its genetic segments although there is no clear evidence of reassortment [23].

We performed characterization of the S, M and L segments of INKV isolated from a mosquito-borne surveillance exercise in northern Sweden, with an aim of investigating the genetic diversity of INKV in northern Sweden. Investigation of the codon usage of INKV and their vertebrate (human) and invertebrate (mosquito) hosts was performed. In the present study, we report the isolation and first whole genome analyses of the mosquito-borne INKV in northern Sweden. This information is vital in understanding the virusadaptation to vectors and hosts considering that arboviruses are utilizing both insect and mammalian cell machinery for replication.

## Methods

### Study area

**Fig. 1:**
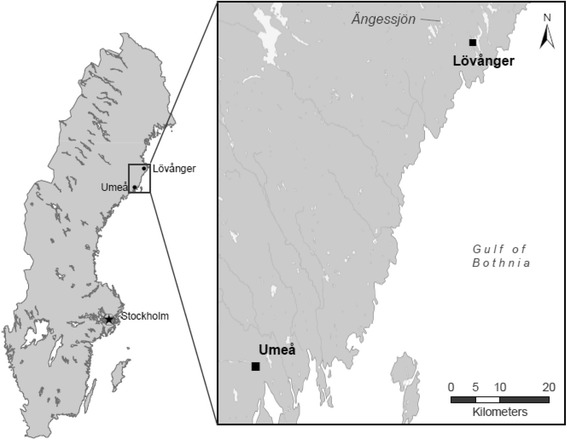
A map showing the study areas where mosquitoes were sampled between June and September 2015 in Umeå and Lövånger, Västerbotten County, Sweden

This study was conducted between June and September 2015, in selected areas of Västerbotten County, northern Sweden, see Fig. 1. Three sites were selected in the Umeå area by the virtue of their close proximity to the inlet of the Gulf of Bothnia and the mouth of Umeå River containing many natural bird habitats. The geographic coordinates for the three sites (1, 2 and 3) in Umeå were: N 63° 44,925’ E 020° 17,718’; N 63° 45,661’ E 020° 18,458’) and N 63° 45,086’ E 020° 20,764’ respectively. Although the climate is subarctic, the area experiences fairly warm summers, which provide a conducive environment for diverse mosquito species to thrive. In addition, Umeå is the largest city in northern part of Sweden with about 120,000 inhabitants, and serves as the port where ferry lines connect to Vaasa city in Finland. Umeå is situated along the “Blue highway” that link Sweden by international traffic to Norway, Finland and Russia.

Two sites were selected in Lövånger due to a previous outbreak of Ockelbo disease that occurred in 2013 [24]. The exact positions of the two sites (4 and 5) in Lövånger were: N 64° 24,635’ E 021° 12,569’ and N 64° 24,530’ E 021° 12,951’ respectively. These sites are close to the lake Ängessjön where SINV, the causative agent of Ockelbo disease, were previously isolated from samples of captured mosquitoes [10].

### Mosquito sampling and processing

Mosquitoes were collected every second week from June–September using propane mosquito magnets (Acreto, Gothenburg, Sweden). The mosquitoes were gently removed from the nets and sorted into 50 mL tubes according to date, site and tube number before stored at −80 °C until processing.

### Homogenization and pooling

The mosquitoes were homogenized individually in 550 μL Dulbecco’s Modified Eagles Media (DMEM, Sigma-Aldrich, St Louis MO, US), 2% HEPES (Fisher Scientific, Fair Lawn, NJ, US). About 60 μL of ten individual mosquito homogenates (1×) were pooled to produce 10× pools (60 μL from 10 mosquito homogenates = 600 μL of 10× pool), and similarly, 60 μL from ten 10× mosquito homogenates were pooled together to make 100× pools (60 μL from ten 10× mosquito homogenates = 600 μL of 100× pool). The preparation of samples was performed at +4 °C, and subsequently stored at −80 °C.

### Extraction of viral nucleic acid

Extraction of viral nucleic acid was performed by Viral NA Extraction Kit using the magnetic bead based method (DiaSorin, Dublin, Ireland). The procedure was performed according to the manual (total Viral NA 94, DiaSorin, Ireland), with 250 μL of the homogenate and 10 μL of protein kinase K (Qiagen, Hilden, Germany) in 1.5 mL sterile micro-centrifuge tubes. Afterwards, viral nucleic acid was extracted and collected in an elution volume of 50 μL.

### Reverse transcriptase-polymerase chain reaction (RT-PCR) and gel electrophoresis

RT-PCR was performed on the nucleic acid extracts with *Orthobunyavirus* genus-primers (Eurofins Genomics, Ebersberg, Germany) see Additional file 1: Table S1 [25, 26]. For *Orthobunyavirus* positive samples, INKV specific PCR was also performed, using primers for S-, M- and L-segments (Eurofins Genomics) see Additional file 2: Table S2. For every PCR reaction, 12.5 μL reaction mix, 5 μL of template, 0.5 μL of both forward and reverse primers, 0.5 μL of a combination of SuperScript™ III Reverse Transcriptase (RT) and Platinum™ Taq DNA Polymerase (Invitrogen, Carlsbad, CA, US) and 6 μL water was used. The PCR was performed after an initial cDNA synthesis and denaturation at 55 °C, 30 min and 94 °C, 2 min; and continued with 94 °C, 15 s, 60 °C, 30 s and 68 °C, 1 min for 45 cycles, with a final extension at 68 °C for 5 min. About 10 μL of the PCR products were mixed with 6× loading dye and analyzed by gel electrophoresis, in 1× TAE buffer and 1.2% agarose gels with added GelRed (Biotium Inc. Hayward, CA, US). Positive 100× pools were further examined by screening the related 10× pools and individual mosquito pools (1×) using the same procedure.

### Purification of RT-PCR products for sequencing

INKV positive RT-PCR-products were purified using QiaQuick purification kit (Qiagen, Hilden, Germany) and ethanol precipitation. Purified samples were sent to Eurofins Genomics (Germany) for sequencing with INKV specific primers, see Additional file 2: Table S2. The sequences of the open reading frames of the S, M and L-segments of INKV strain Lövånger are stored in GenBank under the accession numbers KX554935, KX554936 and KX554937, respectively.

### Virus isolation

Samples of 100× homogenates, including all INKV RNA positive pools were added to BHK-21 and Vero B4 cells. Both cell lines were cultured in 24-well plates at 37 °C and 5% CO_2_. Briefly, 50 μL of homogenate was inoculated on confluent 24-well plates seeded with Vero B4 and BHK-21 cells. The inoculated plates were incubated for one hour before adding 1 mL of pre-heated media and observed once per day for cytopathic effects. The media composition for Vero B4 cells included; DMEM supplemented with 2% fetal bovine serum (FBS) (GE Healthcare Life Sciences, Cramlington, UK), 2% HEPES, 2% penicillin streptomycin (PEST) (GE Healthcare Life Sciences, South Logan, UT, US) and 2% L-glutamine, whereas that of BHK-21 contained; Glasgow MEM supplemented with 5% FBS, 1.3 g/L tryptose (Difco^TM^; Becton, Dickinson and Company, Sparks Glencoe, MD), 2% HEPES, 1 mM sodium pyruvate, 2% PEST and 2% L-glutamine.

### Amino acid substitution and phylogenetic analyses

Virus sequences obtained in the study were identified by BLAST (Basic Local Alignment Search Tool, www.ncbi.nlm.nih.gov/blast) against GenBank databases. The sequences obtained from the study (S segment-KX554935, M segment- KX554936 and L-segment KX554937) segments were aligned to the respective segment sequences of the Finnish prototype strain KN3641 using Muscle. Amino acid substitution analysis of the INKV S, M and L-segment was performed using Muscle programme incorporated within MEGA6 [27]. Phylogenetic trees were constructed from nucleotide alignments using the Maximum Likelihood method based on the Tamura-Nei model [28]. Evolutionary analyses were conducted in MEGA6 [27].

### Amplification of the cytochrome oxidase subunit I (COI) gene

DNA extraction of individual INKV positive mosquito homogenates was performed using Nucleospin Tissue extraction kit (Macherey-Nagel, Düren, Germany). The extracted DNA was amplified using qPCR as described before [12]. Primers used for COI gene amplification were; GB_1358_83F, 5′-ACTCAAGAAAGAGGTAAAAAGGAAAC-3′ and TL2-N-3014R, 5′-TAATATGGCAGATTAGTGCATTGGA-3′. The PCR amplification was performed by KAPA SYBR FAST qPCR Kit KAPA Biosystems, Boston, MA, USA). The preferred cycling conditions were as follows; enzyme activation at 95 °C for 3 min followed by 40 cycles of denaturation at 95 °C for 3 s, annealing and extension at 60 °C for 30 s. A melting curve was produced to evaluate the amplification. The PCR products were visualized on 1.2% agarose gels containing GelRed (Biotium, Inc.). The PCR products were purified by ethanol precipitation and sequenced by Eurofins Genomics. The obtained sequences were compared with available sequences in GenBank to determine their species identity.

### Codon usage and G/C-content

In order to investigate the G/C content of the different segments of the Inkoo virus genome we used the Endmemo software http://www.endmemo.com/bio/gc.php. Codon usage was monitored using http://www.bioinformatics.org/sms2/codon_usage.html.

## Results

### Virus detection in collected mosquitoes

Approximately 125,000 mosquitoes were sampled from the indicated sites. Of these, a representative selection of 10,000 mosquitoes that were distributed equally between sampling sites and dates was selected. Four of the one hundred (100×) pools were positive for *Orthobunyavirus* RNA see Table 1. Additional screening of the corresponding 10× mosquito pools and individual mosquito pools (1×), indicated that three mosquitoes, caught in Lövånger site 4 were *Orthobunyavirus* RNA positive pools and later, when using INKV specific primers, demonstrated to be INKV. To confirm the viability of INKV positive 100× pools, inoculation of the corresponding samples on BHK-21 and Vero B4 cells was done. Two of the 100× pools showed a clear cytopathic effect in culture and were further verified as INKV by RT-PCR and sequencing.

**Table 1:** Inkoo virus positive pools according to period, location and geographic position

100× INKV positive pools	Month/Year	Location, site	Geographic position
1	June 2015	Lövånger, 4	N 64°.24.6’ E 21°.12.5’
2	June 2015	Lövånger, 4	N 64°.24.6’ E 21°.12.5’
3	August 2015	Umeå site, 2	N 63°.45’ E 20°.18’
4	July 2015	Umeå site, 3	N 63°.45’ E 20°.20’

### Mosquito species determination

The COI gene of the three INKV positive individual mosquito homogenates was amplified for species identification. The findings indicated that all positive INKV individual mosquito homogenates were identified as *Ae. communis*. However, the source of the isolates from the 100× pools may not be known due to the pooling method used.

### Amino acid substitution and phylogenetic analyses

**Fig. 2:**
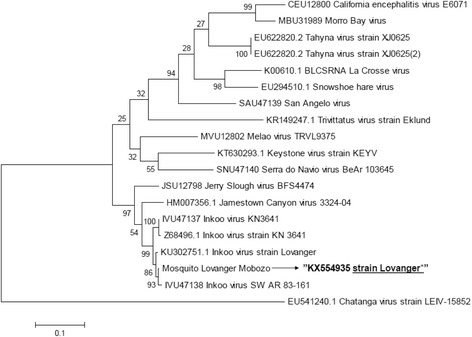
Phylogenetic analysis of the INKV S segment in comparison to 17 other viruses belonging to the California serogroup using the Maximum Likelihood method based on the Tamura-Nei model [20]. The new isolate INKV strain from Lövånger (indicated in bold) clustered most closely with INKV strains Finland, Jamestown Canyon virus and Jerry Slough virus. There were a total of 1,104 positions in the final dataset. Evolutionary analyses were conducted in MEGA6 [19]. Accession numbers; California encephalitis virus (E6071) U12800.1, Morro Bay virus (U31989.1), Tahyna virus strain (XJ0625), Tahyna virus strain XJ0625 (EU622820.2), La Crosse virus (K00610.1), Snowshoe hare virus (EU294510.1), San Angelo virus VR723 (U47139.1), Trivittatus virus strain Eklund (KR149247.1), Melao virus TRVL9375 (U12802.1), Keystone virus strain KEYV (KT630293.1), Serra do Navio virus BeAr 103645 (U47140.1), Jerry Slough virus BFS4474 (U12798.1), Jamestown Canyon virus strain 3324-04 (HM007356.1), INKV Prototype KN3641 (U47137.1), INKV strain KN3641 (Z68496.1), INKV strain Lövånger, larvae (KU302751.1), the study isolate INKV Lövånger, mosquito (KX554935), INKV SWAR 83-161 (IVU47138.1), and Chatanga virus strain LEIV-15852 EU541240.1 respectively

**Fig. 3:**
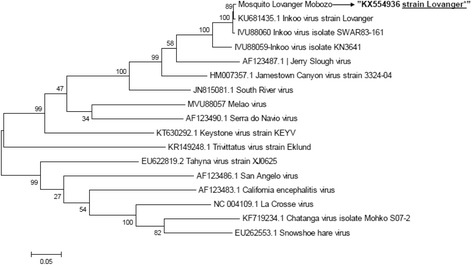
Phylogenetic analysis of the INKV M segment in comparison to 16 other viruses belonging to California serogroup. The M segment (KX554936) differed most from other INKV isolates, but most closely related to INKV Lövånger strain (KU681435), previously isolated from *Ae. communis* larvae in Västerbotten. The evolutionary history was inferred by using the Maximum Likelihood method based on the Tamura-Nei model [22]. There were a total of 4,612 positions in the final dataset. Evolutionary analyses were conducted in MEGA6 [21]. The study isolate is indicated in bold. Accession numbers; the study isolate INKV Lövånger strain (KX554936), Inkoo virus strain Lövånger (KU681435.1), Inkoo virus isolate SWAR83-161 (IVU88060), Inkoo virus KN 3641 (IVU88059), Jerry Slough virus (AF123487.1), James Canyon virus (HM007357.1), South River virus (JN815081), Melao virus (MVU88057), Serra do Navio virus (AF123490.1), Keystone virus strain KEYV (KT630292.1), Trivittatus strain Eklund (KR149248.1), Tahyna virus strain XJ0625 (EU622819.2), San Angelo virus (AF123486.1), Carlifornia encephalitis virus (AF123483.1), La rosse virus (NC004109.1), Chatanga virus isolate Mohko S07-2 (KF719234.1) and Snowshoe hare virus (EU262553.1) respectively

Amino acid substitution analysis of the S (KX554935), M (KX554936) and L (KX554937) segments of the present INKV isolate, in comparison with the Finnish prototype strain KN3641 strain isolated from *Ae. communis,* indicated both synonymous and non-synonymous mutations in all three RNA segments). The S segment showed two non-synonymous and 14 synonymous mutations in the coding sequence of the nucleocapsid gene and non-structural (NSs) gene. Interestingly, the M segment presented 46 non-synonymous mutations and 234 synonymous mutations in contrast to the much longer L segment that had only 18 non-synonymous mutations among the 381 nucleotide changes. Thus, the M-segment had the highest frequency of non-synonymous mutations, 10/1000 bases, compared to 2/1000 and 3/1000 bases for the S- and L-segment, respectively. A phylogenetic analysis indicated that the S segment (KX554935) was closely related to previous INKV isolates as well as Jamestown Canyon virus (JCV) (Fig. 2). The M segment (KX554936) differed most from other INKV isolates, but was most closely related to the INKV Lövånger strain (KU681435), previously isolated from *Ae. communis* larvae in Västerbotten, Sweden, as well as to JCV (Fig. 3). The L segment (KX554937) was close to the INKV KN3641 strain (EU789573.1) isolated from *Ae. communis* in Finland. Both isolates were identical in sequence.

### Codon usage and G/C content

**Fig. 4:**
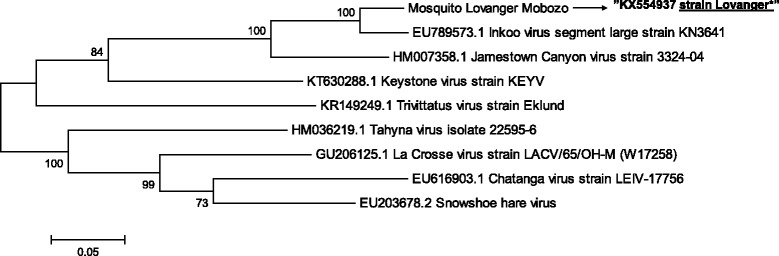
Phylogenetic analysis of the INKV L segment in comparison to 8 other viruses belonging to California serogroup, accession number; EU789573.1, HM007358.1, KT630288.1, KR149249.1, HM036219.1, GU206125.1, EU616903.1 and EU203678.2. The evolutionary history was inferred by using the Maximum Likelihood method based on the Tamura-Nei model [20]. There were a total of 7004 positions in the final dataset. Evolutionary analyses were conducted in MEGA6 [19]. The study isolate is (KX554937) indicated in quotes

The distinctive codon usage patterns of INKV was analyzed (Fig. 4). The G/C contents of the genes in the individual INKV encoded segments were relatively low; S-segment (43.9%), M-segment (35.6%) and the INKV L-segment recorded the lowest G/C content (33.8%) (Table 2).

**Table 2:** G/C content of INKV genes in the respective segments

Virus	S-segment (%) N/NSs genes	M-segment (%) Polyprotein gene	L (%) Polymerase gene
Inkoo Lövånger (mosquito)^a^	43.9/45.5	35.6	33.8
Inkoo virus SW AR 83–161^b^	43.8/45.2	35.5	ND
Inkoo Lövånger (larvae)^c^	43.2/45.2	35.6	ND
Inkoo KN3641^d^	42.5/44.8	35.6	33.6

ND - No sequence; ^a^INKV isolated from adult mosquitoes in Lövånger area; ^b^INKV isolated in Sweden in 1983; ^c^INKV isolated from mosquito larvae in Lövånger area; ^d^INKV isolated from Finland which is the Finnish prototype strain

## Discussion

The European continent, in comparison to other continents such as Africa and Asia, has so far experienced a lower activity of mosquito-borne viruses, possible due to its temperate climate conditions. This may also be linked to the limited diversity and abundance of virus competent mosquito species. However, viruses belonging to *Togaviridae* (SINV), *Bunyaviridae* (e.g. Tahyna virus and INKV) and *Flaviviridae* (e.g. West Nile virus) are present in Europe [2, 3]. Nonetheless, there is a dire need for focused surveillance to gain further knowledge on abundance, circulation, transmission and diversity of mosquito-borne viruses, and their corresponding vectors. In this study, mosquitoes sampled from northern Sweden were confirmed to be INKV positive. This finding is consistent with our previous study when INKV was isolated from mosquito larvae of the same region indicating transovarial transmission [10]. It is plausible that INKV positive larvae might have developed into infected adult mosquitoes.

Phylogenetic analyses of the S, M and L segments of the obtained INKV isolate showed that it was most closely related to the other three INKV strains presently available in genetic databases. This finding implied that the INKV strains, including the study isolate, might have a similar genetic background and evolution history. However, more genetic data on INKV is needed to support this hypothesis.

The present INKV sequence included numerous non-synonymous mutations, especially in the M-segment of the study isolate, in comparison to prototype KN3641 strain, isolated from *Ae. communis* in Finland, which points toward the existence of diverse INKV strains. The high genetic variation of in total nearly 50 amino acids could in the long run have impact on virus infectivity, host range and adaptation to environmental conditions.

When comparing the S, M and L segments of this study, the M segment showed a 3–5 times higher frequency of non-synonymous mutations, in comparison to the S- and L segments. This may be attributed to the presence of conserved sequence motifs located close to the transcription termination site of the S-segment [29, 30] and the conservative nature of the RNA dependent polymerase gene of the L segment, which is known to have several catalytically important motifs containing conserved amino acid regions [19, 31]. In addition, the presence of the large number of synonymous mutations may imply that the RNA dependent polymerase gene is under negative selection pressure that slows down the evolution process since the non-synonymous mutation rate was relatively low.

The actual drivers of codon usage bias among RNA viruses are not known. However, nucleotide base composition (G/C content) and codon usage are shown to vary amongst different organisms, for example G/C content in prokaryotes ranges between 0.25 to 0.75 whereas that of vertebrates is between 0.3 to 0.65 [32, 33]. This variation may be dependent on mutation pressure, differences in codon usage and translational selection. Tick-borne *Flaviviruses* have been shown to have lower G/C content compared to other *Flaviviruses* transmitted by mosquitoes and vertebrate hosts [34]. In the current study, the INKV G/C content was observed to be comparatively low. This is consistent with a study by Jenkins and Holmes, (2003) who analyzed sequences of approximately 50 RNA viruses for codon usage bias and found out that their overall codon usage bias was low with very slight variations amongst their genes [35]. However, in our study, variation in the G/C contents was observed within the different INKV genes (Table 2).

The identification of *Ae. communis* as the species associated with all three INKV positive mosquito individuals confirmed its role as a key vector for INKV [7]. *Ae. communis* is the most common and widely distributed mosquito species in northern Sweden [11], and is frequently found in other parts of Sweden as well [12, 36, 37]. Genetic barcoding of mosquitoes by sequencing the COI gene is useful for determining the species of potential viral vectors, and their future implication in the transmission and dissemination of arboviruses [12].

## Conclusions

In conclusion, the study provides additional evidence on *Aedes communis* as the principal vector of INKV. The sequence data obtained from this study will aid in building up of the scanty genetic data available in the sequence archives. These findings clearly highlight the importance of surveillance for early detection and response to new potential threats and when designing appropriate prevention and control strategies. The study was limited to northern Sweden and therefore the findings may not be a true reflection of the actual scenario in other parts of the country. Though the pooling approach employed in the study saved the cost incurred in purchasing reagents and supplies, it was not sensitive enough to allow detection of lower copies of the virus since it created a dilution effect. Also, it was difficult to design appropriate broad-range primers targeting all the viruses within the California serogroup.

## Additional files

Additional file 1: Table S1. Sequences of *Orthobunyavirus* genus specific primers used for RT-PCR. (PDF 586 kb)

Additional file 2: Table S2. Sequences of INKV specific primers used for RT-PCR and sequencing. (PDF 187 kb)

## Abbreviations

BHK: Baby hamster kidney
COI: Cytochrome oxidase subunit I
HEPES: 4-(2-hydroxyethyl)-1-piperazineethanesulfonic acid
INKV: Inkoo virus
SINV: Sindbis virus
