# Supplementary material for: Mosquito-borne Inkoo virus in northern Sweden - isolation and whole genome sequencing
Source: Virol J. 2017 Mar 23;14:61. doi: 10.1186/s12985-017-0725-5 (PMC5362992; doi:10.1186/s12985-017-0725-5)
Supplement: Supplementary file 1 — Sequences of Orthobunyavirus genus specific primers used for RT-PCR. (PDF 586 kb) [file 12985_2017_725_MOESM1_ESM.pdf]

Table S1. Sequences of Orthobunyavirus genus specific primers used for RT-PCR

Primer	Sequence
OrthoBun For	5’-CTGCTAACACCAGCAGTACTTTTGAC -3’
OrthoBun Rev	5’-TGGAGGGTAAGACCATCGTCAGGAACTG -3’
BCS82C	5’-ATGACTGAGTTGGAGTTTCATGATGTCGC -3’
BCS332V	5’-TGTTCCTGTTGCCAGGAAAAT -3’
