# Supplementary material for: Mosquito-borne Inkoo virus in northern Sweden - isolation and whole genome sequencing
Source: Virol J. 2017 Mar 23;14:61. doi: 10.1186/s12985-017-0725-5 (PMC5362992; doi:10.1186/s12985-017-0725-5)
Supplement: Supplementary file 2 — Sequences of INKV specific primers used for RT-PCR and sequencing. (PDF 187 kb) [file 12985_2017_725_MOESM2_ESM.pdf]

Table S2. Sequences of INKV specific primers used for RT-PCR and sequencing

**S-segment**	
INKV S1F	5′-AGTAGTGTGCTCCACTGAATACATTTAA-3′
INKV S600R	5′-ACTGAGGATCCATCATACCATGCTT-3′
INKV S400F	5′-GATGACGATGAGTCCCAAAGAGAA-3′
INKV S988R	5′-GTAGTGTGCTCCACTGAATACATTTAA-3′
**M-segment**	
INKV M1F	5′-AGTAGTGTACTACCAAGCATAGAAAACGTTCA-3′
INKV M1065R	5′-TTCACCAATGACCATAGCACTAATAGGA-3′
INKV M941F	5′-CCTCAGAGCAGCCAGAGTTATGT-3′
INKV M2049R	5′-CGGGCCATACTTTCTGCAATATATGAA-3′
INKV M1728F	5′-CGAATATGCCTTCCTTACTAGATACTG-3′
INKV M3086R	5′-CCTATTACATCCATTAGGTAGTTGCCC-3′
INKV M2957F	5′-TGGAAGGTATTGAAACTGTTGAAGGGATA-3′
INKV M4067R	5′-TTGTGTTACAGATCTTAAATTTTAGAGACA-3′
INKV M3545F	5′-CTAGGTGTGTACTCCAAAGGATGTG-3′
INKV M4507R	5′-GTAGTGTGCTACCAAGTATATTTAAATGA-3′
**L-segment**	
INKV L3F	5′-CCGAGTAGTGTACTCCTATTTACA-3′
INKV L470R	5′-AAATCACGGCTAATAGGATCTATCC-3′
INKV L67F	5′-ACAGAATATCAACAATTCCTTGCTCG-3′
INKV L 1059R	5′-TATAGATTGTAGTGACTTTGATAA-3′
INKV L891F	5′-GGAAGGCTGGGAGATGATGGT-3′
INKV L 1889R	5′-AAAAGACCAGGAGAGGATACTATTC-3′
INKV F2005	5′-GAACCTGCACGCTATATGATAATGAA-3′
INKV L 12868R	5′-CATCATATCCATAATTAATTCTATAGYGG-3′
INKV F3229	5′-AATGCAGATATGTCTAAATGGAGTGC-3′
INKV L 14066R	5′-AAACACCATTCAATTCTATTGGTATATCT-3′
INKV F4273	5′-GATGCTGAAATGGACCCAAGTGA-3′
INKV L 14952R	5′-TTACTATATGCTCTAAGTACTAATGCAGG-3′
INKV F4920	5′-TTCTCCTGCATTAGTACTTAGAGCATA-3′
INKV L 15937R	5′-CTGGTTCTTATTGCCATATGTTCTTCATT-3′
INKV F5728	5′-AAGACACCAGAAAATATATCAATAAGTGG-3′
INKV L 6922R	5′-GTTATATTTTTAATTTGAATATGGCCAATT-3′

a) Nucleotide code: Y = Pyrimidine (C or T)
